# An Assessment of Knowledge, Attitude, and Practice (KAP) of Colorectal Cancer among Community Pharmacists in the Qassim Region of Saudi Arabia

**DOI:** 10.3390/pharmacy12020042

**Published:** 2024-02-27

**Authors:** Mohammed Alshammari, Saleh Al-Maktoum, Abdulrahman Alsharidah, Abubakar Siddique, Mohammed Anaam, Saud Alsahali, Yasser Almogbel, Ali Alkhoshaiban

**Affiliations:** Department of Pharmacy Practice, College of Pharmacy, Qassim University, Buraidah, Qassim 51452, Saudi Arabia; m.alshammari@qu.edu.sa (M.A.); s.almaktoum@qu.edu.sa (S.A.-M.); 382123559@qu.edu.sa (A.A.); getabu2u@gmail.com (A.S.); m.anaam@qu.edu.sa (M.A.); y.almogbel@qu.edu.sa (Y.A.); askhshieban@qu.edu.sa (A.A.)

**Keywords:** colon cancer, colorectal cancer, knowledge, attitude, practice, assess, screening

## Abstract

**Background**: The global burden of colorectal cancer remains a major public health issue and one of the leading causes of death worldwide. In Saudi Arabia, it continues to be a health concern. Any delays in diagnosis for any reason may contribute to advanced complications; therefore, pharmacists’ knowledge and awareness of colorectal cancer are crucial for the welfare of society. Studies of colon cancer-related knowledge, attitude, and practice (KAP) among community pharmacists have not previously been conducted in the Al-Qassim region of Saudi Arabia. In the present study, therefore, we sought to investigate the KAP on colon cancer among pharmacists in Al-Qassim. **Methods:** This was a prospective, cross-sectional, observational study. A sample of 150 community pharmacists was recruited using a convenience sampling method. A self-administered questionnaire was used to evaluate levels of knowledge and practice. **Results:** Out of a total of 150 pharmacists, the majority of respondents (60.7%) possessed an adequate level of knowledge. About 50% of participants had heard of the early screening test, and 68.7% knew that colonoscopy is necessary in such scenarios. On the basis of their attitudes, 41.3% of study participants were aware of colon cancer symptoms and risk factors. In practice, however, the majority of pharmacists (81%) did not perform early cancer screenings, while 19% did screen when advised to do so by a physician. **Conclusions**: Our results indicate that pharmacists in Qassim have an adequate level of knowledge of colon cancer in terms of awareness, assessment, and screening. Since community pharmacists are among the most reliable members of the medical community, a greater awareness of colon cancer among pharmacists may improve public knowledge of the disease.

## 1. Introduction

Colorectal cancer, also known as colon cancer, is the third-most prevalent form of cancer in developed countries and ranks among the leading causes of death worldwide [1]. Researchers in developed nations have reported increased rates of the disease. In addition, due to population growth and an increase in the aging population, the incidence of colorectal cancer is expected to increase in the coming years [1,2]. One recent estimate reported by the Saudi Cancer Registry suggests that the incidence of colon cancer among the Saudi population will increase by 60% by 2030. In Saudi Arabia, colon cancer is now the most common cancer after breast cancer. It ranks first among men, at 9.9%, and third among women, at 6.4% [3].

In the United States, excluding skin cancers, colon cancer ranks third in terms of incidence. According to the American Cancer Society, 106,180 new diagnoses of colon cancer were expected in the United States in 2023 [4]. In the UK, between 2016 and 2018, about 120 colorectal cancer cases were diagnosed every day, resulting in approximately 42,900 new cases annually [5]. Several studies have indicated that early screening reduces colon cancer mortality [6].

In Saudi Arabia, the majority of patients are diagnosed at advanced stages with metastases. At such stages, therapy is challenging, and mortality rates are high. A better awareness of early symptoms could potentially help to eliminate risk factors such as having a sedentary lifestyle, being overweight, consuming a diet high in fat and low in fiber, having polyps in the colon lining, being of advancing age, and smoking, all of which are associated with an increased risk of colon cancer [7,8].

The role of community pharmacists in society is significant, as they participate in screening for prostate, colon, and cervical cancer. Today, it is possible to conduct cancer screenings at community pharmacies. Screening helps reduce mortality by identifying cancer at the early stages of the disease. Screening activities involve a variety of methods, such as test kits, questionnaires, and patient education. By identifying those at higher risk of cancer, such activities are an excellent way to increase participation in screening programs. As the prevalence of CRC rises, involving pharmacists in health promotion efforts becomes crucial for elevating public awareness and knowledge. Empowering community pharmacists to circulate the correct information and engage in educational campaigns can significantly contribute to enhancing CRC awareness and prevention within the community and acting as a proactive approach to colorectal health [9].

Improvements in the knowledge of pharmacists and a better public awareness of the screening services that they provide will help to ensure high-quality patient care and identify areas for further improvement with respect to early signs and symptoms and risk factors. As a result, individuals within at-risk population groups are more likely to be assessed and referred to hospitals for early diagnosis, thus raising treatment success rates. The current situation is due to a lack of protocols emphasizing the role of the community pharmacist in screening and prevention programs [9].

Researchers have long hypothesized that a majority of individuals are unaware of colorectal cancer and diagnostic procedures for the disease [10,11]. In addition, a number of studies have indicated that perception and behavior with respect to screening are inadequate among populations at high risk [12,13]. Researchers have examined low screening-participation rates. A lack of knowledge among pharmacists and a poor attitude toward cancer screening in general have been identified as major problems [14,15].

In this study, therefore, we sought to evaluate knowledge, attitude, and practice on colorectal cancer among community pharmacists in the Qassim region of Saudi Arabia, specifically with respect to assessment and screening. With regard to screening in particular, it appears that pharmacists in the Qassim region have low levels of awareness and that this situation is likely to continue, at least for the foreseeable future [16].

Patients frequently consult pharmacists for health advice because pharmacists have historically been regarded as among the most reliable members of the medical community. Furthermore, pharmacists may play a useful role in contacting hard-to-reach populations, including those of lower socioeconomic status who may be at higher risk of cancer but who are presently less likely to be screened [17,18].

As with many other forms of cancer, colon cancer survival rates increase significantly when it is diagnosed early and treated effectively. However, previous research has yielded inadequate results in terms of raising public awareness and, more specifically, educating community pharmacists. Such pharmacists have a vital role to play in raising public awareness of colon cancer and helping to prevent the disease [19,20].

So far as we are aware, no study has previously been conducted in the Qassim region of Saudi Arabia that has specifically examined the involvement of pharmacists in the early detection of colorectal cancer (CRC).

## 2. Materials and Methods

### 2.1. Study Design and Location

A prospective, cross-sectional observational study was conducted to determine the KAP on colon cancer among community pharmacists in the Qassim region of Saudi Arabia over a period of four months.

### 2.2. Data Collection and Sampling

Data collection was performed between March 2023 and July 2023 using a structured and validated online questionnaire. Responses to the questionnaire were self-assessed and self-reported. The community pharmacies in the five cities of the Qassim region in Saudi Arabia were approached via conventional sampling by data collectors. Prior to the start of this study, the data collectors visited participating pharmacists in order to clarify the objectives of the study. Participants were recruited from a number of community pharmacies in rural and urban areas in the Qassim region.

In the current study, we employed a conventional sampling method to recruit participants. This involved engaging data collectors who visited pharmacies that were conventionally selected. The process continued until we successfully obtained our target sample size of 150 participants. In case a selected pharmacy declined to participate, we promptly moved on to the next one until we reached our desired sample size.

### 2.3. Development of the Questionnaire

A structured online questionnaire was obtained from a previous study. Permission was obtained from the author of that study, and expert validation was further obtained. The questionnaire was based on previous KAP research, which was modified to meet the needs of the present work [21].

The questionnaire consisted of questions, categorized into three sections, as follows: demographics of the participants; pharmacists’ knowledge of colon cancer; and pharmacists’ attitude and practice with respect to colorectal cancer.

In the first part of the questionnaire, there were seven items that focused on demographic and general information relating to participants. The following part contained 24 questions on knowledge of colon cancer and it’s early screening. The third part included two questions concerning the practice of community pharmacists in relation to colon cancer and three questions focusing on their attitude toward the disease. This part of the questionnaire also assessed the sources of information used by participants (e.g., WHO, colleges, TV, or MOH).

Answers to questions about attitude were evaluated using a Likert scale (strongly agree, agree, neutral, disagree, and strongly disagree). Some items in the questionnaire were framed using the two possible answers ‘Yes’ and ‘No’. A score of one was given for a correct answer and a score of zero for an incorrect answer. Answering ‘I don’t know’ or ‘I’m insured’ was scored as an incorrect answer.

Based on previous research, using Bloom’s cut-off point criteria [22], the following levels of awareness, attitude, and practice were determined: moderate (60 to 79%); high (80% or above); and low (less than 60%).

### 2.4. Data Analysis

After being taken from Google forms, the data were coded and entered into version 25.0 of the Statistical Package for Social Sciences (SPSS) (SPSS Inc., Chicago, IL, USA) program. Descriptive statistics were produced for the study variables. Pearson’s chi-squared tests and t-tests were carried out for statistical analysis among nominal variables. A one-way analysis of variance (ANOVA) test was performed when the subject of comparison was present in all three groups. An independent samples t-test was conducted when the subject of comparison was present. Statistical significance was determined by considering differences to be significant if the *p*-value was less than 0.05. For standardization, we expressed the mean sums of scores as percentages.

### 2.5. Ethics Considerations

Participation in this study was completely voluntary. Participants were free to refuse to take part in this study. The research ethics committee of Qassim University in Qassim, Saudi Arabia, approved this study (approval date: 22 March 2023; number of certifications of approval: 23-30-11).

## 3. Results

### 3.1. Demographics

In our study, a total of 150 pharmacists completed the survey, which assessed their knowledge, attitude, and practice of colorectal cancer.

The distribution of participants’ attributes is presented in the next section. Table 1 below summarizes respondents’ characteristics based on independent factors (age group, education, etc.). We found that 57.3% of study participants were aged 25 to 34 years, 24% were aged 35 to 44 years, and 11.3% were aged 45 to 55 years. In addition, 6.7% of participants were less than 25 years old. Participants were predominantly male, with men making up 75.3% of the survey group and women only 24.7%. The great majority of participants (92%) were qualified college graduates, while 5.3% possessed a diploma, and only three participants had a master’s degree. In terms of years of experience, 32.7% had fewer than three years of employment, 26.7% had between four and six years, 17.3% had between seven and nine years, and 23.3% had ten years or more. Concerning location, 34.7% of study participants were from Buraidah, 27.3% from Unizah, 15.3% from Al-Rass, 12% from Al-Mithnab, and 10% from Al-Badayea. A total of 61.3% of pharmacists worked in chain pharmacies, while 38.7% worked in non-chain pharmacies. A majority of study participants (85.3%) reported no family history of colon cancer, while 14.7% did report a family history of the disease.

As shown in Table 1, the only statistically significant relationship that we found was between knowledge and the home region of participants (*p* < 0.05), i.e., participants from Buraidah had better knowledge of colorectal cancer than participants from other regions.

### 3.2. Knowledge

The mean total knowledge score was 14.4 ± 3.6 out of the maximum attainable score of 24. None of the participants attained a high level of knowledge (≥80%). As shown in Table 2, half of the participants (50%) had not heard about the early cancer screening test. Most items in the questionnaire related to general information about colorectal cancer that pharmacists were adequately aware of, such as risk factors and symptoms of the disease. A majority of participants (78.7%) were aware that colon cancer could be prevented.

As can be seen in Table 3, the most important sources for information on colon cancer were social media, the Ministry of Health (MOH), booklets, and the WHO, which were used by 53.3%, 47.3%, 45.3%, and 40.7% of respondents, respectively.

### 3.3. Attitude

One of the aims of this study is to study the attitude of pharmacists toward CRC, following their attitudes towards CRC. About 80.7% of pharmacists positively answered the attitude questions. The results revealed that 41.4% of pharmacists thought that they were not susceptible to colon cancer (mean = 2.67, SD = 1.008). A total of 61.3% of study participants thought that they had adequate information about CRC (mean = 3.21, SD = 0.950). However, 70.0% of pharmacists thought that elderly people were more susceptible to colon cancer, with an average response (mean = 3.81, SD = 0.944), as shown in Table 4. In general, the community pharmacists in the sample have positive attitude toward CRC as shown in Table 5. 

### 3.4. Practice

Another aim of this study was to evaluate the practice of CRC screening among community pharmacists. The total practice score was 2, while the mean score was 0.36 ± 0.68. The results revealed that 12.7% of pharmacists have performed early checkups for colon cancer (mean = 0.13, SD = 0.334). A total of 23.3% of study participants thought about undergoing a screening for early detection of colon cancer (mean = 0.23, SD = 0.424), as shown in Table 6. However, a t-test revealed that there was a statistically significant difference in the mean practice score between genders and in terms of family history (*p* < 0.05). On the other hand, the t-test analysis failed to show any statistically significant difference in the mean practice score between groups in terms of community pharmacy types (*p* > 0.05).

A one-way ANOVA revealed that there was a statistically significant difference in the mean practice score between the following demographics: age (F (4, 145) = [4.990]), education levels (F (3, 146) = [15.276]), years of experience (F (3, 146) = [2.811]), and regions of work (F (4, 145) = [6.183] (*p* =< 0.05). There was no correlation between knowledge and practice, as well as between knowledge and attitude.

## 4. Discussion

As colorectal cancer is one of the most prevalent cancers among the population of Saudi Arabia, this study was conducted on community pharmacists in Qassim, Saudi Arabia. This study targeted community pharmacists due to their prominence in society, their ease of access to health care providers, their work, and because they are considered one of the most visited by the population. This study mainly focused on early symptoms, risk factors, and screening methods.

Our results revealed that most respondents had an adequate level of knowledge. More than half of the study participants thought they had adequate information about colorectal cancer. This finding was in line with the results of a previous study [23]. About 68.7% of pharmacists in the present study had heard about early colorectal cancer screening using colonoscopy, while 56% knew that blood testing was one of the methods used for early detection of colon cancer; however, this was lower than the corresponding figure reported in a previous study [24].

The only statistically significant finding (*p* > 0.05) in the present study concerned knowledge and the home region of participants, i.e., participants from Buraidah had a better knowledge of colorectal cancer. No statistically significant correlations were found between knowledge and educational attainment. Previous studies have reported a lack of association between gender and knowledge of CRC [25]. However, similar to other studies, we found that male CPs had a good knowledge of CRC [26].

The most frequently used sources of information about colorectal cancer among pharmacists were social media, the Ministry of Health (MOH), booklets, and the WHO, which were used by 53.3%, 47.3%, 45.3%, and 40.7% of respondents, respectively. These results are not consistent with those reported in another study, where awareness campaigns, booklets, RAD/TV, and newspapers were used by 22.9%, 22.1%, 18.2%, and 18.2%, respectively [22]. Overall, we found that 41.4% of pharmacists thought that they were not susceptible to colon cancer, while 41.3% thought that they had adequate information about the disease. The percentage of community pharmacists who thought that elderly people were more susceptible to colorectal cancer was 70.0%. In addition, and in line with the results of a previous study, we found that 78.7% of our participants thought that colorectal cancer could be prevented, and 83.3% thought that detection of colon cancer at an early stage increased survival rates [27]. Overall, the attitude of community pharmacists was considered positive, i.e., above 80%, according to Bloom’s cut-off point criteria.

In the present study, 73.3% of participants believed that smoking was a major factor for colorectal cancer, and 64.7% believed that a high-fat and low-fiber diet was a factor; however, only 60.0% of respondents were aware that the presence of polys in the lining of the colon and rectum was one of the factors.

Reducing the main risk factors and adhering to screening recommendations would reduce the incidence of colorectal cancer. Lifestyle risk factors, such as inactivity, obesity, and tobacco use, are increasingly prevalent. For this reason, a new healthcare policy should be executed in public health programs to prevent diseases. According to one study, smoking is the biggest risk factor [6], however, the authors of another study argued that drinking more than one unit of alcohol per day represents the biggest risk factor [26]. Colorectal cancer is indicated by symptoms that may be local, such as increased mucus secretion in the stool, or systemic, such as pain and cramps in the stomach. Most of our participants (72.0%) were aware of the symptom, the presence of blood in stool, and a majority (66.7%) were aware that a sudden change in the number of bowel motions and diarrhea was also a symptom. In addition, 62.7% of our participants believed that unexpected weight loss was a sign of colon cancer, a finding in line with results from previous investigations [28]. Among participants in the present study, the great majority did not perform early screenings for colon cancer; indeed, only 19.7% of respondents did so. However, 23.3% of pharmacists intended to carry out screenings in the future or at least thought about doing so. These results are also compatible with findings previously published in the literature [29]. The low incidence of screenings for colorectal cancer may be due to several factors, including a shortage of time, the complexity of scheduling a visit to a doctor, a low understanding of screening, and individual concerns. A lack of national policies that promote screening might also be a factor [30].

Saudis who are at average risk for colorectal cancer but show no signs should be screened. The American Cancer Society (ACS) recommends that colonoscopies be carried out, beginning at age 45 for those in the general population and at age 35 for those individuals at higher risk of cancer. Because social media was found to be the most frequently used informational tool in our study, it is important to emphasize education using social media in the future. Doing so will lead to increased survival rates in cases of colorectal cancer that are detected early and also enhance the prevention of the disease.

Despite our efforts to measure the KAP of pharmacists on CRC, our study had some limitations. It is possible that the administration of the questionnaire as part of an internal audit process affected the integrity and honesty of participant responses. Also, this study was carried out exclusively in the Qassim region of Saudi Arabia and targeted only community pharmacists, so the results could not be generalized to other regions or populations. Furthermore, the conventional sampling used in this study may have a greater chance of selection bias. Since the participants were recruited from certain pharmacies, the sample may not be fully representative of the entire population of interest.

## 5. Conclusions

In this study, we found that community pharmacists in the Qassim region of Saudi Arabia have adequate knowledge of colon cancer. However, earlier studies have revealed a lack of such knowledge among the general Saudi population. Because community pharmacists are among the most reliable members of the medical profession, they may be used to improve public knowledge of colorectal cancer. To accomplish this, national screening programs, annual educational campaigns, and, most importantly, since it is easily accessible and a source of information, social media should be utilized effectively.

## Figures and Tables

**Table 1:** The extent of pharmacists’ knowledge of colorectal cancer based on their characteristics.

Variables	N 150 (100%)	Low (39.3%)	Moderate (60.7%)	*p* Value
**Age group (years)**				
<25	10 (6.7)	5 (50.0)	5 (50.0)	
25–34	86 (57.3)	31 (36.0)	55 (64.0)	
35–44	36 (24.0)	15 (41.7)	21 (58.3)	
45–55	17 (11.3)	8 (47.1)	9 (52.9)	
>55	1 (0.7)	0 (0.0)	1 (100.0)	
				*0.506*
**Gender**				
Male	113 (75.3)	46 (40.7)	67 (59.3)	
Female	37 (24.7)	13 (35.1)	24 (64.9)	
				*0.961*
**Education**				
Diploma	8 (5.3)	4 (50.0)	4 (50.0)	
Bachelor	138 (92.0)	53 (38.4)	85 (61.6)	
Master	3 (2.0)	1 (33.3)	2 (66.7)	
Doctorate	1 (0.7)	–	1 (100)	
				*0.107*
**Years of experience**				
0–3	49 (32.7)	18 (36.7)	31 (63.3)	
4–6	40 (26.7)	17 (42.5)	23 (57.5)	
7–9	26 (17.3)	8 (30.8)	18 (69.2)	
10 years or more	35 (23.3)	16 (45.7)	19 (54.3)	
				*0.536*
**Region**				
Unaizah	41 (27.3)	15 (36.6)	26 (63.4)	
Buraidah	52 (34.7)	18 (34.6)	34 (65.4)	
Al–Rass	23 (15.3)	12 (52.2)	11 (47.8)	
Al–Badayea	16 (10.7)	7 (43.8)	9 (56.3)	
Al–Mithnab	18 (12.0)	7 (38.9)	11 (61.1)	
				*0.026* *
**Type of community pharmacy**				
Chain	92 (61.3)	31 (33.7)	61 (66.3)	
Non-chain	58 (38.7)	28 (48.3)	30 (51.7)	
				*0.574*
**Family history of colorectal cancer**				
Yes	22 (14.7)	9 (40.9)	13 (59.1)	
No	128 (85.3)	50 (39.1)	78 (60.9)	
				*0.462*

* Significance (*p* < 0.05).

**Table 2:** Knowledge of colorectal cancer and its early screening *(n* = 150).

Variables	Frequency of ‘Yes’	Percentage
**Knowledge**		
Have you ever heard of early cancer screening test?	75	50.0
Can colon cancer be prevented?	118	78.7
Do you think colon cancer is fatal?	105	70.0
Do colon cancer recovery rates increase when detected in early stages?	125	83.3
**Methods of early detection of colon cancer**		
Colonoscopy	103	68.7
PR speculum	74	49.3
Blood detection in the stool sample	71	47.3
Barium dye for large intestine	78	52.0
Blood tests	84	56.0
Abdominal CT scan	77	51.3
Clinical examination of the rectum	67	44.7
**Risk factors for colon cancer**		
Men and women are susceptible to colon cancer	65	43.3
Physical inactivity	72	48.0
Overweight	89	59.3
High-fat low-fiber diet	97	64.7
Presence of polyps in the lining of the colon and rectum	90	60.0
Increasing age	86	57.3
Smoking	110	73.3
**Symptoms of colon cancer**		
Presence of blood in the stool	108	72.0
Sudden weight loss	94	62.7
Increases mucus secretions in the stool	79	52.7
Feeling that the rectum is not fully emptied with defecation	97	64.7
Pain and cramps in the stomach	86	57.3
Sudden change in number of bowel motions and diarrhea	100	66.7

**Table 3:** Sources of information used by respondents for screening for colorectal cancer.

Sources	No.	Percentage (%)
Social media	80	53.3
MOH	71	47.3
Booklets	68	45.3
WHO	61	40.7
Doctors	60	40.0
Colleagues	56	37.7
Radio/TV	39	26.0
Newspapers	35	25.3
Awarness campaign	35	23.3

MOH—Ministry of Health; WHO—World Health Organization.

**Table 4:** Pharmacists’ attitudes towards CRC in detail (*n* = 150).

Items	SA	A	N	D	SD	Mean	SD *
I think I am susceptible to colon cancer	9	13	66	43	19	2.67	1.008
	6.0%	8.7%	44.0%	28.7%	12.7%		
I think I have adequate information	9	53	54	28	6	3.21	0.950
	6.0%	35.3%	36.0%	18.7%	4.0%		
Elderly people are more susceptible to colon cancer	34	71	32	9	4	3.81	0.944
	22.7%	47.3%	21.3%	6.0%	3.7%		
Overall						9.69	1.904

SA—strongly agree; A—agree; N—neutral; D—disagree; SD—strongly disagree; SD *—standard deviation.

**Table 5:** Pharmacists’ overall attitude measure (*n* = 150).

Attitude towards CRC	No.	%
Positive	121	80.7
Negative	29	19.3
Total	150	100%

**Table 6:** Pharmacists’ practice towards CRC (*n* = 150).

Practice towards CRC	Frequency of Yes. (%)	Frequency of No.	Mean	SD
Have you done early checkups for colon cancer?	19 (12.7)	131 (87.3)	0.13	0.334
Have you ever thought about undergoing a screening for early detection of colon cancer?	35 (23.3)	115 (76.7)	0.23	0.424
		Total	0.36	0.688

## Data Availability

The datasets used for this study are available from the corresponding author on reasonable request.
